# The Structure of the Membrane Protein of SARS-CoV-2 Resembles the Sugar Transporter SemiSWEET

**DOI:** 10.20411/pai.v5i1.377

**Published:** 2020-10-19

**Authors:** Sunil Thomas

**Affiliations:** 1 Lankenau Institute for Medical Research, Wynnewood, PA-19096, USA

**Keywords:** SARS-CoV-2, COVID-19, Coronavirus, Virus, Sugar transporter, SemiSWEET, Membrane glycoprotein, Pandemic

## Abstract

**Background:**

Severe acute respiratory syndrome coronavirus 2 (SARS-CoV-2) is responsible for the disease COVID-19 that has decimated the health and economy of our planet. The virus causes the disease not only in people but also in companion and wild animals. People with diabetes are at risk of the disease. As yet we do not know why the virus has been highly successful in causing the pandemic within 3 months of its first report. The structural proteins of SARS include membrane glycoprotein (M), envelope protein (E), nucleocapsid protein (N), and the spike protein (S).

**Methods:**

The structure and function of the most abundant structural protein of SARS-CoV-2, the membrane (M) glycoprotein, is not fully understood. Using *in silico* analyses we determined the structure and potential function of the M protein.

**Results:**

The M protein of SARS-CoV-2 is 98.6% similar to the M protein of bat SARS-CoV, maintains 98.2% homology with pangolin SARS-CoV, and has 90% homology with the M protein of SARS-CoV; whereas, the similarity is only 38% with the M protein of MERS-CoV. *In silico* analyses showed that the M protein of SARS-CoV-2 has a triple helix bundle, forms a single 3-trans-membrane domain, and is homologous to the prokaryotic sugar transport protein SemiSWEET. SemiSWEETs are related to the PQ-loop family whose members function as cargo receptors in vesicle transport, mediate movement of basic amino acids across lysosomal membranes, and are also involved in phospholipase flippase function.

**Conclusions:**

The advantage and role of the M protein having a sugar transporter-like structure is not clearly understood. The M protein of SARS-CoV-2 interacts with S, E, and N protein. The S protein of the virus is glycosylated. It could be hypothesized that the sugar transporter-like structure of the M protein influences glycosylation of the S protein. Endocytosis is critical for the internalization and maturation of RNA viruses, including SARS-CoV-2. Sucrose is involved in endosome and lysosome maturation and may also induce autophagy, pathways that help in the entry of the virus. Overall, it could be hypothesized that the SemiSWEET sugar transporter-like structure of the M protein may be involved in multiple functions that may aid in the rapid proliferation, replication, and immune evasion of the SARS-CoV-2 virus. Biological experiments would validate the presence and function of the SemiSWEET sugar transporter.

## INTRODUCTION

The coronavirus disease 2019 (COVID-19) is currently responsible for the pandemic that has decimated the health and economy of every country. COVID-19 is regarded as a respiratory disease that manifests with fever, cough, shortness of breath or difficulty breathing, chills, muscle pain, headache, sore throat, and loss of taste and smell. Other symptoms include diarrhea, nausea, and vomiting [1, 2]. Many patients with the COVID-19 are asymptomatic but are able to transmit the virus to others [3, 4]. The prolonged pandemic has resulted in social distancing, travel restrictions, decreased trade, high unemployment, commodity price decline, and financial stress that has impacted the global economy. COVID-19 disease is caused by the severe acute respiratory syndrome coronavirus 2 (SARS-CoV-2), a member of the betacoronavirus genus [5]. The disease has resulted in a mortality of 0.5% to 8.0%. Several factors influenced the death rate in people with COVID-19. Age, health, and behavior of the population impacted the death rate due to COVID-19. Old people, people with underlying diseases such as diabetes, lung diseases (due to smoking), liver disease, cardiovascular disease, and obesity are more prone to death due to COVID-19. As yet, there are no effective drugs available for treatment of the disease nor vaccines available commercially to protect against the virus.

The major structural proteins of SARS-CoV-2 are spike (S), membrane (M), envelope (E), and the nucleocapsid (N) proteins [6, 7]. The spike protein of SARS-CoV-2 uses the host angiotensin-converting enzyme 2 (ACE2) as the entry receptor [8]. Hence, the research community has an interest in studying the spike protein for drug and vaccine development. Amraie *et al* [9] recently reported that the C-type lectin receptors CD209L/L-SIGN and CD209/DSIGN serve as alternative receptors for SARS-CoV-2 entry into human cells. The C-type lectin domain could function as a calcium-dependent glycan-recognition domain.

The most abundant structural protein of coronaviruses is the M glycoprotein; it spans the membrane bilayer, leaving a short NH2-terminal domain outside the virus and a long COOH terminus (cytoplasmic domain) inside the virion [10]. The M protein can bind to all other structural proteins. Binding with M protein helps to stabilize N proteins and promotes completion of viral assembly by stabilizing the N protein-RNA complex, inside the internal virion [11]. As the M protein cooperates with the S protein, mutations may influence host cell attachment and entry of the viruses [12]. The S protein of the virus is glycosylated and this modification may aid in immune evasion [13, 14]. However, it is not known how the S protein is glycosylated. The function of the M protein is also not fully understood.

Sugars Will Eventually be Exported Transporters (SWEETs) and SemiSWEETs are sugar transporters in eukaryotes and prokaryotes, respectively. SWEET proteins were first identified in plants as a novel family of sugar transporters that mediates the translocation of sugars across cell membranes [15–18]. Sugar transporters are essential for the maintenance of blood glucose levels in animals, nectar production, phloem loading, seed and pollen development in plants, and also in pathogen nutrition [15, 18]. Engineering of SWEET mutants using genomic editing tools has been shown to mediate resistance to pathogens [19].

In eukaryotes, SWEET can discriminate and transport the uptake of mono and disaccharides across the plasma membrane by allowing solutes to permeate across biological membranes following a concentration gradient [15, 19, 20]. Eukaryotic SWEETs are composed of 7 transmembrane helices (TMHs) that contain a pair of 3 transmembrane repeats, which are connected by an additional helix, while SemiSWEETs, the homologues of SWEETs in prokaryotes, contain 3 TMHs [16, 21]. The human genome contains only 1 *SWEET* gene and may be involved in glucose transport [15].

The prokaryotic SemiSWEETs may be involved in the metabolism and transport of sugar synthesis. The SemiSWEETs of prokaryotes are more diverse than SWEETs in plants; they seldom have homologues sharing >50% identity [17]. The limited number of SemiSWEET homologues suggest that they are not as important as the SWEETs in eukaryotes [17].

The function and role of the M proteins of the SARS-CoV-2 during host infection is not clearly understood. Here, we report that the M proteins of SARS-CoV-2 are structurally similar to Semi-SWEET sugar transport proteins of prokaryotes based on *in silico* analyses.

### Materials and Methods

#### SARS-CoV-2 protein structure

The structural protein sequences of the SARS-CoV-2 were downloaded from the Pubmed (https://www.ncbi.nlm.nih.gov/pubmed) protein database. The structural proteins include Membrane protein (Accession No. QJA17755), Envelope protein (Accession No. QJA17754), Spike protein (Accession No. QHR63290), and Nucleocapsid protein (Accession No. QJC20758).

#### Protein modeling

Three-dimensional (3-D) structures of proteins provide valuable insights into their function on a molecular level and inform a broad spectrum of applications in life science research. A detailed description of the interactions of proteins and the overall quaternary structure is essential for a comprehensive understanding of biological systems, how protein complexes and networks operate, and how they could be modulated. SWISS-MODEL is a server that is used for 3-D structure prediction. SWISS-MODEL is the first fully automated protein homology modeling server and is updated continuously [22]. In our study, homology modeling was constructed using the SWISS-MODEL server (http://swissmodel.expasy.org/) and the iterative threading assembly refinement (I-TASSER) (https://zhanglab.ccmb.med.umich.edu/I-TASSER/) with default settings. The M protein sequence of SARS-CoV-2 was entered in FASTA format.

Residue-based diagrams of proteins, also called snake diagrams or protein plots, are 2-D representations of a protein sequence that contain information about properties such as secondary structure [23]. To determine a snake diagram model of a protein we used Protter (http://wlab.ethz.ch/protter). Protter is an interactive and customizable web-based application that enables the integration and visualization of both annotated and predicted protein sequence features together with experimental proteomic evidence for peptides and posttranslational modifications onto the transmembrane topology of a protein. It allows users to choose from numerous annotation sources, integrate their own proteomics data files, select the best-suited peptides for targeted quantitative proteomics applications, and export publication-quality illustrations [24].

#### Sequence alignment

Multiple sequence alignments (MSAs) are essential in most bioinformatics analyses that involve comparing homologous sequences [25]. ClustalW2 is a server for MSA that is also used for phylogenetic tree analysis. Multiple sequence alignments between the M protein of SARS-CoV-2 and the M proteins of SARS-CoV, bat SARS-CoV, pangolin SARS-CoV, and MERS-CoV, as well as SemiSWEET sequences from different microorganisms, were performed using the ClustalW2 server (http:/www.ebi.ac.uk/tools/msa/clustalW2/).

## RESULTS

The S protein of SARS-CoV-2 binds to ACE2 receptors of the host for cell entry and may be a key target for drugs and vaccines. Hence, the S protein of SARS-CoV-2 virus is well characterized. The SARS-CoV-2 is one of the most successful viruses as it caused a pandemic within just 3 months of its first reported occurrence in Wuhan, China. As yet, we do not know why the virus has been successful in inducing a pandemic leading to millions of infections and thousands of deaths.

Three-dimensional protein structures provide valuable insights into the molecular basis of protein function [26]. Using *in silico* techniques the structure and potential function of the M protein of the SARS-CoV-2 virus was elucidated.

**Figure 1.**
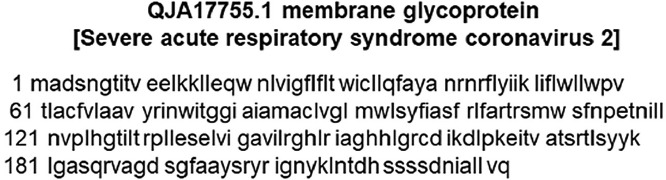
The protein sequence of the M glycoprotein of SARS-CoV-2. The sequence was downloaded from the NCBI protein database.

**Figure 2.**
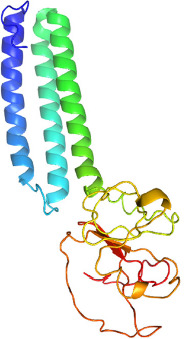
Predicted M protein structure of SARS-CoV-2 (ribbon diagram) using the software I-TASSER.

The structural protein sequence of the membrane protein (M) of SARS-CoV-2 is shown in Figure 1. The FASTA sequence of the M protein was entered into the SWISS-MODEL server and I-TASSER. Based on the sequence, the structure of the molecule was predicted as the bidirectional sugar transporter SWEET2b. The ribbon representation model of the M protein as predicted using I-TASSER is shown in Figure 2.

The sugar transporter SWEETs of eukaryotes are generally composed of 7 transmembrane helices. Modeling proteins using residue-based diagrams (snake diagrams) helps us to understand their function. Hence, we used Protter to model the M protein.

**Figure 3.**
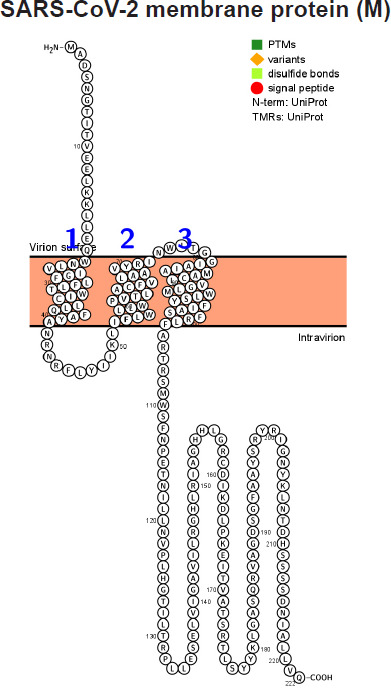

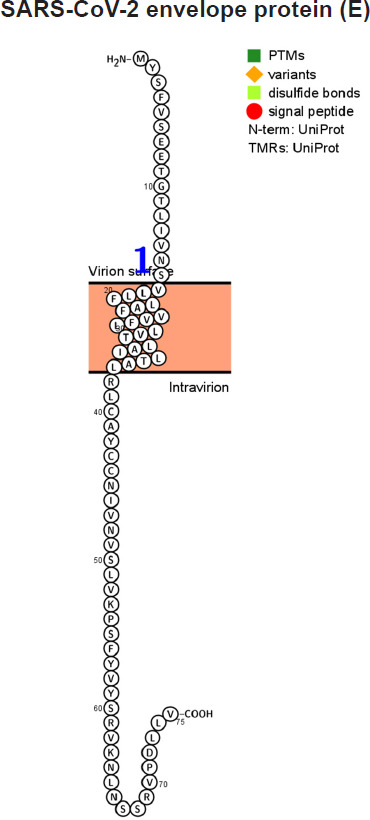

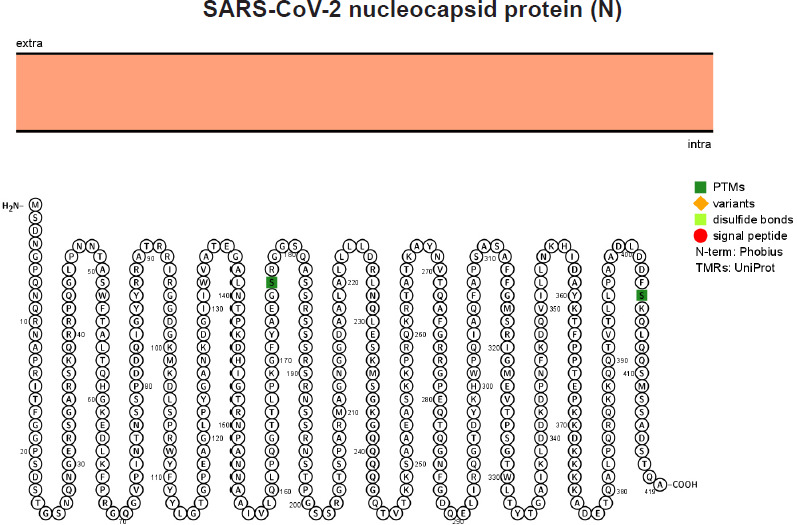

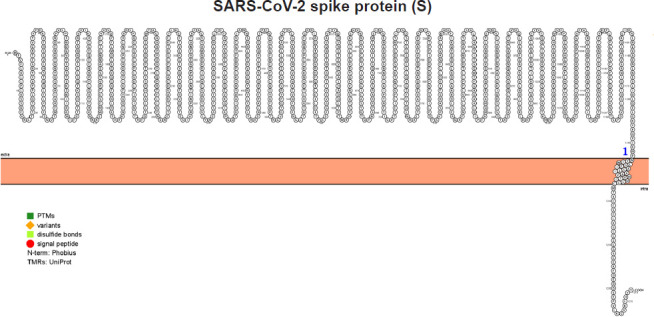
Membrane topology of proteins (snake diagrams) determined using Protter. (A) The membrane (M) glycoprotein of SARS-CoV-2 has a triple helix bundle and formed a single 3-transmembrane domain. (B) Snake diagram of envelope (E) protein, (C) nucleocapsid (N) protein, and (D) spike protein (S).

The M glycoprotein is the most abundant envelope protein of SARS-CoV-2. *In silico* analyses of the M protein of SARS-CoV-2 using Protter demonstrated that it has a triple-helix bundle, and forms a single 3-transmembrane domain. In addition, the M glycoprotein has a short amino terminal domain outside the viral envelope and a long carboxy-terminal domain inside the viral envelope (Figure 3A). The SWISS-MODEL predicted the M glycoprotein as SWEET2b. However, the M protein only has 3 transmembrane helices, not the 6 or 7 transmembrane helices which are observed in the SWEET sugar transporters of eukaryotes. Hence, the M glycoprotein structure of SARS-CoV-2 may be considered as SemiSWEET. To confirm accuracy of the study, we also modeled the E, N, and S proteins of SARS-CoV-2. The modeling showed that the E protein has a short outer amino terminal domain, a single helix, and a long inner carboxy-terminal domain (Figure 3B). The N protein had its entire structure inside the viral envelope (Figure 3C). Whereas, the S protein had the majority of its structure outside the viral envelope and a short carboxy-terminal domain inside the viral envelope (Figure 3D).

**Figure 4.**
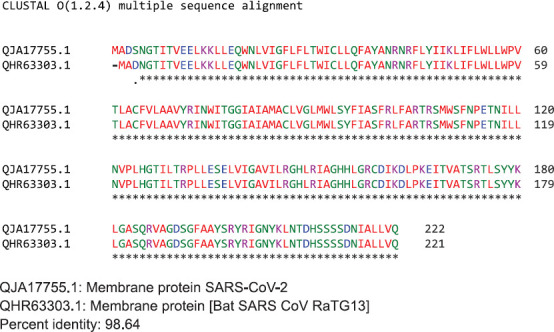

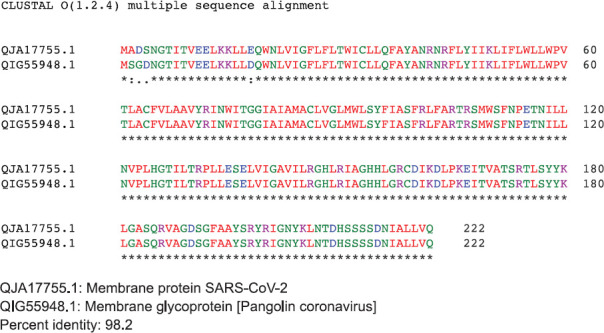

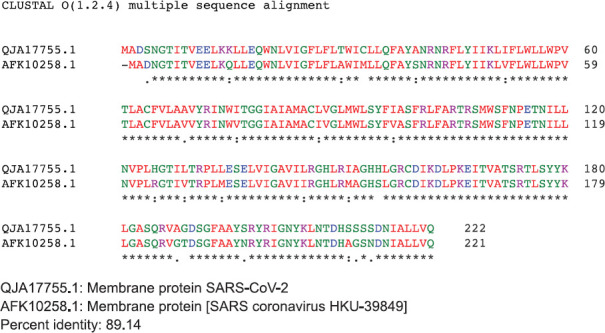

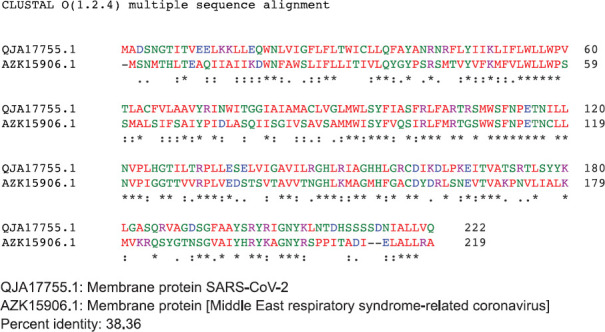
Protein sequences were aligned using ClustalW. Comparison of protein sequence of the M protein of SARS-COV-2 with (A) M protein of Bat SARS-CoV, (B) M protein of pangolin SARS-CoV, (C) M protein of SARS-CoV, and (D) MERS-CoV.

ClustalW2 was used to determine homology between M proteins of different coronaviruses. SARS-CoV-2 M protein has a sequence similarity of 98.6% with the M protein of bat SARS-CoV, 98.2% homology with the pangolin SARS-CoV, 89.14% similarity with the M protein of SARSCoV and a sequence similarity of 38.36% with the M protein of MERS-CoV (Figures 4A-D). The MERS-CoV M protein had more homology with the sugar transporter SWEET (Table 1).

**Table 1.** **Homology of the M protein of SARS-CoV-2 with the M protein of other coronaviruses and the sugar transporter SWEET**

Membrane Protein	Membrane protein identity (with SARS-CoV-2)	Bidirectional sugar transporter SWEET identity
SARS-CoV-2	100	14.3
Bat SARS-CoV	98.64	14.3
Pangolin SARS-CoV	98.2	14.3
SARS-CoV	89.14	14.3
MERS-CoV	38.36	20.0

**Figure 5.**
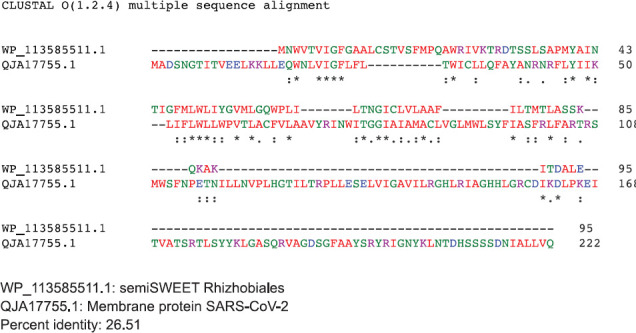

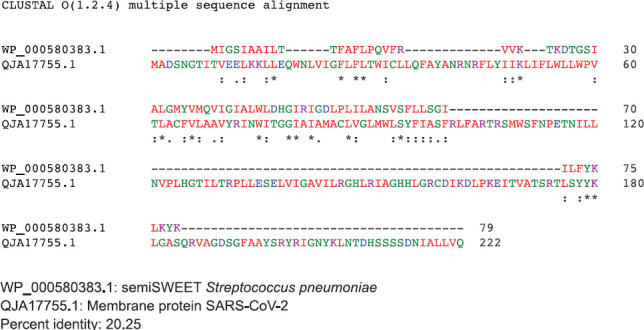
Protein sequences were aligned using ClustalW. (A) Comparison of protein sequence of the M protein of SARS-COV-2 with SemiSWEET sugar transporter of Rhizobiales. (B) Comparison of protein sequence of the M protein of SARS-COV-2 with SemiSWEET sugar transporter of *Streptococcus pneumoniae.*

The SemiSWEET sugar transporters of prokaryotes are more diverse than the SWEET counterparts in plants. In the prokaryotes the SemiSWEET seldom share identity. We used ClustalW2 to determine sequence homology of the sugar transporters of multiple microorganisms. The sequence of SemiSWEET of the M glycoprotein of SARS-CoV-2 had a similarity of 26% with the SemiSWEET of *Rhizobiales* and 20% with *Streptococcus pneumoniae* demonstrating that the SemiSWEET of the SARS-CoV-2 may be highly conserved (Figures 5A, B).

## DISCUSSION

The COVID-19 pandemic caused by the coronavirus SARS-CoV-2 is spreading at an alarming rate and has resulted in an unprecedented health emergency all over the world [27]. The rapid spread of SARS-CoV-2 justifies the global effort to identify effective preventive strategies and optimal medical management [28].

As yet there are no effective vaccines to protect against COVID-19 nor effective approved drugs to treat patients with the disease. The development of antivirals is an urgent priority to combat the disease [27]. In the absence of effective and safe vaccines or antivirals to control the disease, strategies for mitigating the burden of the pandemic are focused on non-pharmaceutical interventions, such as social-distancing, contact-tracing, quarantine, isolation, and the use of face masks in public [29].

The primary route of transmission of COVID-19 is likely via respiratory droplets and is known to be transmissible from pre-symptomatic and asymptomatic individuals [30]. Infected people spread viral particles during talking, breathing, coughing, or sneezing. Such viral particles are known to be encapsulated in globs of mucus, saliva, and water, and the fate/behavior of globs in the environment depends on the size of the globs [31]. Studies show that SARS-CoV-2 can be detected in the air and remain viable 3 hours after aerosolization. The weight of combined evidence supports airborne precautions for the occupational health and safety of health workers treating patients with COVID-19 [32].

It has been shown that wearing a mask reduces the contact transmissibility by reducing transmission of infected droplets in both laboratory and clinical contexts. Public mask-wearing is most effective at reducing the spread of the virus when compliance is high. The decreased transmissibility could substantially reduce the death toll and economic impact while the cost of the intervention is low [30]. The community-wide benefits are likely to be greatest when face masks are used in conjunction with other non-pharmaceutical practices such as social-distancing, and when adoption is nearly universal (nation-wide) and compliance is high [33]. Chu *et al* [34] support physical distancing of 1 meter or more and hypothesized that contact tracing could reduce the disease transmission.

Understanding the biochemical events of the coronavirus replication cycle may provide a number of attractive targets for drug development [27]. Current strategies involve developing drug and vaccine candidates against the spike (S) protein of the virus. The rationale being that neutralizing antibodies against the S protein prevent uptake of the virus via the human ACE2 receptor [35]. The S proteins are highly glycosylated making them targets for carbohydrate-binding agents such as lectins. Liu *et al* [36] showed that the lectin FRIL (Flt3 receptor-interacting lectin), isolated from the hyacinth bean (*Lablab purpureus*), has anti-SARS-CoV-2 activity. FRIL binds preferentially to complex-type N-glycans and neutralizes viruses that possess complex-type N-glycans on their envelopes. FRIL could effectively neutralize SARS-CoV-2, preventing viral protein production and cytopathic effect in host cells. These data suggest a potential application of FRIL for the prevention and/or treatment of COVID-19 [36]. Identifying drug targets that blunt the activity of the virus may lead to effective treatments for COVID-19.

Viruses are non-living entities, without any organelles and devoid of their own metabolism, though they have the capability to dramatically modify the host cellular metabolism upon entry. Viruses upregulate consumption of glucose and converge on similar metabolic pathways for anabolism [37]. Virus-induced metabolism may provide free nucleotides for rapid viral genome replication, increased amino acid production for rapid virion assembly, and high amounts of ATP for the high energy costs of genome replication and packaging. The mechanism for increased glucose uptake by the virus is still not clearly understood.

Glucose is the energy source of cells and tissues. Cellular uptake of glucose is a fundamental process for metabolism, growth, and homeostasis. Glucose is a polar molecule that does not readily diffuse across the hydrophobic plasma membrane of cells. Glucose molecules are transported through the glucose transporters that include GLUTs, the sodium-driven glucose symporters SGLTs, STP, and SWEETs [38]. SWEETs are seen in plants and animals. SWEET induction by plant pathogens leads to secretion of sucrose that is used by these microorganisms for nutrition/reproduction [39].

The bacterial ancestors of SWEET, known as SemiSWEET are the smallest of the sugar transporters and assemble into dimers [21, 40, 41]. In fact, eukaryotic SWEETs consist of 2 Semi-SWEET-like units fused via an inversion linker transmembrane helix [17]. The diverse gene neighbors of SemiSWEETs suggest that SemiSWEETs may transport diverse substrates and play several physiological roles in different organisms [17]. The SWEETs and their bacterial homo-logues, SemiSWEETs, are related to the PQ-loop family, characterized by highly conserved pro-line and glutamine residues (PQ-loop motif) [41]. The PQ-loop family exhibits diverse activities; they function as cargo receptors in vesicle transport, mediate movement of basic amino acids across lysosomal membranes, and are also involved in phospholipase flippase function [42–44]. As yet, there are no reports of sugar transporters in viruses.

It is not known how SARS-CoV-2 has been successful in spreading all over the world within 3 months of its first reported occurrence in Wuhan, China. Identifying the mechanisms of how viruses alter cellular metabolism and where in the virus life cycle these metabolic changes are necessary will provide an understanding of virus replication needs and potentially provide cellular targets for inhibition of these viruses. In this paper using *in silico* data analysis we demonstrate that the structure of the membrane (M) glycoprotein of SARS-CoV-2 resembles the SemiSWEET sugar transporter of the prokaryotes.

Clues to the viral metabolism can be understood from the patient population at risk of infection. It is known that people with diabetes are more prone to COVID-19 disease [45]. Recent reports indicate that the SARS-CoV-2 induces diabetes in non-diabetic people [46]. A large case study of COVID-19 patients reported that those with diabetes had a 3-fold higher mortality rate than did those without diabetes (7.3% vs 2.3%) [47].

Diabetes is a risk factor and is also prevalent in patients infected with other coronaviruses, including SARS-CoV [48] and Middle East Respiratory Syndrome coronavirus (MERS-CoV) [49, 50]. It has been demonstrated that SARS coronavirus enters pancreatic islets and damages islets causing acute diabetes [48]. As people with diabetes have high glucose, the environment may favor proliferation of viruses. MERS-CoV utilizes dipeptidyl peptidase 4 (DPP4), and modeling of the structure of SARS-CoV-2 spike glycoprotein predicts that it can interact with human DPP4 in addition to ACE2. The protein DPP4 is a ubiquitous membrane-bound aminopeptidase that circulates in plasma; it is multifunctional with roles in nutrition, metabolism, and immune and endocrine systems. DPP4 activity differentially regulates glucose homeostasis and inflammation via its enzymatic activity and nonenzymatic immunomodulatory effects. DPP4 inhibitors, or gliptins are approved for the treatment of type 2 diabetes mellitus [51]. Rhee *et al* [52] reported that DPP-4 inhibitor is significantly associated with a better clinical outcome of patients with COVID-19.

A virus uses multiple mechanisms for the uptake of glucose. Human cytomegalovirus (HCMV), a herpesvirus, induces the sugar transporter, GLUT4 to increase glucose uptake during infection [53]. Whereas, transmissible gastroenteritis virus (TGEV), a coronavirus, induces multiple sugar transporters EGFR, SGLT1, and GLUT2 for glucose uptake [54]. Rhinoviruses (RVs) are responsible for the majority of upper airway infections, and they enhance the expression of the PI3K-regulated glucose transporter GLUT1; glucose deprivation from medium and via glycolysis inhibition by 2-deoxyglucose impairs viral replication [55].

Sucrose is used for energy metabolism by cells. In addition, sucrose is used for endosome and lysosome maturation, autophagosomes, and also to induce autophagy [56, 57]. Coronaviruses, including SARS and SARS-CoV-2 use endosomes for cellular entry, and they are known to manipulate autophagosomes and autolysosomes for viral dissemination in the cell [58, 59].

The membrane (M) glycoprotein is the most abundant envelope protein of coronaviruses [60]. *In silico* analysis demonstrated that M protein of SARS-CoV-2 is 98.6% similar to the M protein of bat SARS-CoV, maintains 98.2% homology with pangolin SARS-CoV, and 90% homology with the M protein of SARS-CoV; whereas, the similarity is only 38% with the M protein of MERSCoV. Thus, the M protein of SARS-CoV-2 resembles the M protein of bat and pangolin SARSCoV to a greater extent than MERS-CoV. A recent paper by Zhang *et al* [61] reported that at the genomic level SARS-CoV-2 is 96.2% homologous to bat SARS-CoV (RaTG13) and 91.02% homologous to pangolin SARS-Co-V.

*In silico* analysis showed that the M protein of SARS-CoV-2 resembles the sugar transporter, SWEET. Upon analysis, it was observed that other coronaviruses including SARS-CoV, bat SARSCoV, pangolin SARS-CoV, and MERS-CoV have M proteins homologous to the sugar transporter SWEET. Further analysis by residue-based structure demonstrated that the protein has the characteristic structure of SemiSWEET, the sugar transporter of prokaryotes. To our knowledge this is the first report of the presence of a sugar transporter-like structure in a virus membrane. It is known that the prokaryotes have diverse sugar transporters. In our analysis, the SARS-CoV-2 sequence of SemiSWEET has no homology to other prokaryotes.

Generally, the enveloped viruses, including SARS-CoV-2, use a 2-step procedure to release their genetic material into the cell: 1) They bind to specific surface receptors of the target cell membrane, and 2) they fuse the viral and cell membranes. This second step may occur at the cell surface or after internalization of the virus particle by endocytosis [62]. Currently, it is not known how the M proteins of the virus are fused to the host cell membrane. If the M proteins are fused to the host cell membrane, they could theoretically function as a sugar transporter.

An advantage of the virus having a sugar transporter in its membrane is that it may influence energy metabolism. How the virus utilizes sugar molecules is unknown. The SARS-CoV-2 virus may use sugar for multiple purposes. The S protein is highly glycosylated. It could be hypothesized that the sugar transporter-like structure of the M protein influences glycosylation of the S protein. In addition, it could be hypothesized that the sugar transporter-like structure of the virus membrane may influence sucrose entry into the endosome, lysosome, or autophagosome that are manipulated by the virus, thereby aiding the virus release into cells. Thus, the presence of a SemiSWEET glucose transporter in the M protein of the virus may be an efficient mechanism that may induce rapid viral proliferation and immune evasion.

In many infectious diseases caused by either viruses or bacteria, pathogen glycoproteins play important roles during the infection cycle, ranging from entry to successful intracellular replication and host immune evasion [63]. *Toxoplasma gondii* is an intracellular bacteria that transitions from acute infection to a chronic infective state in its intermediate host via encystation, which enables the parasite to evade immune detection and clearance. The tissue cyst perimeter is highly and specifically decorated with glycan modifications that are influenced by *Toxoplasma* nucleotide-sugar transporter (TgNST1). *Toxoplasma* strains deficient for the TgNST1 gene (Δnst1) form cyst-like structures *in vitro* but no longer interact with lectins, as these strains are deficient in the transport and use of sugars for the biosynthesis of cyst-wall structures. The study demonstrated the role of parasite glycoconjugates in the persistence of *Toxoplasma* tissue cysts [64].

People with diabetes are at risk of COVID-19 infection which may be due to the high proliferation of the virus because of unmetabolized glucose. A characteristic of some COVID-19 patients is coagulopathy [65]. Anticoagulant therapy with low molecular weight heparin led to a better prognosis in severely ill COVID-19 patients who were associated with high mortality [66]. Platelets, produced by the megakaryocytes of the bone marrow are responsible for blood clotting. Glucose is taken up by the platelets, mediated through the glucose transporters GLUT1 and GLUT3. Lack of glucose transporters in the platelets reduces platelet counts and increases clearance of platelets [67]. Normal glucose levels reduce platelet activation; whereas, hyperglycemia increases platelet glucose metabolism thereby contributing to increased platelet activation and thrombosis in animal models of diabetes [68].

Lungs in some COVID-19 patients are not effectively oxygenating the blood (hypoxia), but these patients feel alert and healthy and hardly gasp for breath. Glucose transport is acutely stimulated by hypoxic conditions, and the response is mediated by enhanced function of the facilitative glucose transporters GLUT [69, 70]. Prolonged exposure to hypoxia results in enhanced transcription of the GLUT1 glucose transporter gene, with little or no effect on transcription of other GLUT genes [69].

Several pulmonary disorders are associated with a decrease in alveolar oxygen tension, and alveolar epithelial cells (AEC) exhibit different adaptive mechanisms to cope with oxygen deprivation. Under hypoxia, because of inhibition of oxidative phosphorylation, adenosine triphosphate supply is dependent on the ability of cells to increase anaerobic glycolysis. Hypoxia induces stimulation of Na-independent glucose transport and an increase in 2-deoxyglucose uptake; it also induces the glucose transporter, GLUT1 at both protein and mRNA levels [71]. HIF-1α regulates the activity of glucose transporters, GLUT, that are responsible for glucose uptake. Hypoxia-inducible factors (HIFs) are oxygen-sensitive transcription factors that allow adaptation to hypoxic environments [72]. HIF-1α reduces acute lung injury by optimizing carbohydrate metabolism in the alveolar epithelium [73].

An early characteristic of COVID-19 patients is loss of smell. The glucose receptors are expressed in taste receptor cells [74]. Glucose receptors are expressed in the olfactory bulb and changes to the expression of the receptors may influence olfaction [75]. Whereas, Villar *et al* [76] demonstrated that glucose removal and the inhibition of glycolysis or oxidative phosphorylation inhibits odor detection.

The data described in this paper are based on *in silico* analyses; homology models and similarities with plant and bacterial glucose transporters are not adequate to assign a role of the M protein of the virus to specific host comorbidities such as diabetes. Further biological experiments are required to validate the presence and function of the virus membrane sugar transporter.
